# Combining Different Potato-Associated *Pseudomonas* Strains for Improved Biocontrol of *Phytophthora infestans*

**DOI:** 10.3389/fmicb.2018.02573

**Published:** 2018-10-29

**Authors:** Mout De Vrieze, Fanny Germanier, Nicolas Vuille, Laure Weisskopf

**Affiliations:** ^1^Department of Biology, University of Fribourg, Fribourg, Switzerland; ^2^Institute for Plant Production Sciences, Agroscope, Nyon, Switzerland

**Keywords:** late blight, pseudomonads, *Solanum tuberosum*, consortium, biocontrol, rhizosphere, phyllosphere

## Abstract

Late blight caused by *Phytophthora infestans* is considered as the most devastating disease of potato and is a re-emerging problem worldwide. Current late blight control practices rely mostly on synthetic fungicides or copper-based products, but growing awareness of the negative impact of these compounds on the environment has led to the search for alternative control measures. A collection of *Pseudomonas* strains isolated from both the rhizosphere and the phyllosphere of potato was recently characterized for *in vitro* protective effects against *P. infestans.* In the present study, we used a leaf disk assay with three different potato cultivars to compare the disease inhibition capacity of nine selected *Pseudomonas* strains when applied alone or in all possible dual and triple combinations. Results showed a strong cultivar effect and identified strains previously thought to be inactive based on *in vitro* assays as the best biocontrol candidates. One strain was much more active alone than in combination with other strains, while two other strains provided significantly better protection in dual combination than when applied alone. A subset of five strains was then further selected to determine their mutual influence on each other’s survival and growth, as well as to characterize their activity against *P. infestans* in more details. This revealed that the two strains whose dual combination was particularly efficient were only weakly interfering with each other’s growth and had complementary modes of action. Our results highlight the potential to harness the crop’s native rhizosphere and phyllosphere microbiome through re-assembling strains with differing modes of action into small communities, thereby providing more consistent protection than with the application of single strains. We consider this as a first step toward more elaborate microbiome management efforts, which shall be integrated into global strategies for sustainable control of potato late blight.

## Introduction

Sustainable crop production faces the challenge of maintaining high yields to meet the food requirements of an increasing world population while limiting its own environmental impact. In potato production, the major yield-threatening disease is the oomycete *Phytophthora infestans*, causing late blight (Fry, 2008). In Europe, late blight’s costs, resulting both from yield loss and disease control, have been estimated to over 1 billion Euros per year (Haverkort et al., 2008). In conventional agricultural management, potato late blight, as many other diseases, is controlled by multiple applications of fungicides of varying modes of action during the growing season (Haverkort et al., 2008; Cooke et al., 2011). However, increasing awareness of the negative side effects of synthetic pesticides on environmental and human health has led to growing interest in organically produced food. In organic potato production, growers use copper-based products as alternative to synthetic fungicides to protect their crops from late blight, but since copper is not degradable, it accumulates in the soil and is toxic to the soil fauna (Du Plessis et al., 2005; Eijsackers et al., 2005). Therefore, there is a need for alternative measures to control late blight in an environmentally friendly manner. One such alternative is the use of natural enemies of the disease-causing agent, also termed “biological control” or “biocontrol.” Microbial biocontrol agents such as the bacterium *Bacillus thuringiensis* or the entomopathogenic fungus *Beauveria bassiana* have been successfully applied to control insect pests (Arthurs and Dara, 2018), but only few examples (mostly based on bacteria of the genera *Pseudomonas* or *Bacillus*) exist where such biocontrol strategy was efficient enough against fungal pathogens to lead to product commercialization (reviewed in Velivelli et al., 2014). Beyond these few examples, controlling an aggressive pathogen such as *P. infestans* is a significant challenge, as evidenced by the number of biocompatible treatments that have been tested but did not, or only partially, meet the required level of reproducible efficacy (Dorn et al., 2007; Axel et al., 2012; Alaux et al., 2018).

One possible factor underlying this lack of success might be that *P. infestans* can infect its host plants by different means, e.g., through direct germination of sporangia or release of motile zoospores, both leading to host tissue penetration and mycelial development (Fry, 2008). An efficient biocontrol agent should therefore either induce plant resistance (Syed Ab Rahman et al., 2018) or, when acting directly on the pathogen, inhibit both types of infection routes. Alternatively, using different biocontrol agents each targeting one of the pathogen’s modes of infection could improve biocontrol efficiency. Such approaches based on strain combinations rather than on single strains could not only bring functional complementary as exemplified above for sporangia vs. zoospore-mediated infection, but they could also be useful in providing functional redundancy. Indeed, a key element in the success of a biocontrol agent is the ability to colonize its host, which in turn is influenced by many factors including the resident microbiome (Sylla et al., 2013). Using strain consortia might therefore lead to enhanced protection robustness in the face of varying environmental and genetic (e.g., different crop varieties) conditions. To date, few studies have addressed this question and tested the impact of mixed strains rather than single ones on plant protection against diseases. Among these, Niu and co-workers could protect maize against *Fusarium* by applying a consortium of seven different bacterial species (Niu et al., 2017), while Hu and co-workers obtained significantly better protection of tomato against *Ralstonia*-induced wilt when using a mixture of 8 *Pseudomonas* strains than when applying each strain individually (Hu et al., 2016).

We have previously isolated and characterized the protective potential of single *Pseudomonas* strains isolated from the rhizosphere and phyllosphere of potato against *P. infestans* (Guyer et al., 2015; Hunziker et al., 2015). In the present study, we hypothesized that mixing different *Pseudomonas* strains, which differed in their phylogenetic identity, in their origin of isolation (rhizosphere vs. phyllosphere) and in their emission of volatile (De Vrieze et al., 2015) and non-volatile anti-*Phytophthora* metabolites could increase the disease-inhibiting potential of these strains. Since the discrepancy between *in vitro* activity and *in planta* protection is usually large (Dorn et al., 2007), and since we had recently established a high-throughput assay to screen for inhibition of late blight development on leaf material (Guyer et al., 2015), we started with a leaf disk-based screening rather than an *in vitro* screening procedure. To this end, all possible twofold and threefold combinations of nine selected *Pseudomonas* strains were tested for late blight inhibition on leaf disks of three potato cultivars of varying late blight susceptibility. This first experiment enabled to select five promising strains, which were further analyzed for better understanding of the mechanisms underlying the observed synergetic effects. This was done by characterizing their effect as single strains vs. in dual and triple combinations on the pathogen’s development (both mycelial growth and zoospore release), as well as their growth behavior when inoculated alone or in dual and triple combinations, in order to identify putative synergetic or antagonistic effects.

## Materials and Methods

### Bacterial Strains and Culture Media

Nine *Pseudomonas* strains were selected from a collection of strains isolated from the rhizosphere (R) and from shoots (S) of field grown potato plants, based on their *in vitro* activity against *P. infestans* (Guyer et al., 2015; Hunziker et al., 2015). The bacteria were routinely grown on Luria Bertani medium (LB), which was prepared by dissolving 20 g L^-1^ of Difco LB broth (Lennox, United States) in distilled water supplemented with 15 g L^-1^ of agar (Agar-agar, ERNE surface AG, Switzerland). For bacterial cell suspensions, cultures were prepared by suspending single colonies from 2-day-old plates in 0.9% NaCl and streaking the obtained suspensions on LB agar medium. After overnight incubation at 20°C, bacterial cells were resuspended in 0.9% NaCl. For bacterial competition assays, rifampicin-resistant derivative strains obtained as described in Guyer et al. (2015) were used. LB and Pseudomonas Isolation Agar (PIA), supplemented or not with rifampicin (50 μg mL^-1^) and nystatin (500,000 UL^-1^) were used for these experiments. PIA medium was prepared by dissolving 45 g L^-1^ of Pseudomonas Isolation Agar (Fluka) in distilled water, to which 20 mL L^-1^ of glycerol (Sigma–Aldrich) was added. Optical density (OD) measured at 570 nm was used to quantify and adjust bacterial density. The nine strains showed slightly different cell numbers/OD_570_ unit but these differences were within the same order of magnitude: most strains had between 1.4 × 10^8^ and 2 × 10^8^ cells per mL for an OD_570_ of 1, while slightly higher cell numbers were observed for R32, S34 and S35 (between 4.5 × 10^8^ and 5.5 × 10^8^ cells per mL at OD_570_ of 1).

### *Phytophthora infestans* and Culture Media

*Phytophthora infestans* isolate Rec01 (originally isolated by H. Krebs, Agroscope) was used for all inhibition assays and grown on unclarified V8 (10%) medium for collection of sporangia and zoospores, or on Pea agar medium for mycelial growth assays. Unclarified V8 medium was prepared by diluting V8 juice (100 mL L^-1^) amended with CaCO_3_ (1 g L^-1^) and 15 g L^-1^ of agar according to Miller (1955). Pea agar medium was prepared by skimming 120 g of autoclaved frozen peas in water and adding 15 g of agar. The isolate was regularly inoculated on potato slices for host passage. Sporangia suspensions were prepared by scraping off the mycelium of 14-day-old plates and suspending it in demineralized water. After vigorous shaking, the suspension was filtered using cloth to discard the mycelium. Concentration of sporangia was determined using a Thoma chamber. Sporangia suspensions were maintained in the dark until use. To obtain zoospore suspensions, sporangia were subjected to a cold shock by adding ice-cold water to sporangia suspensions in Eppendorf tubes, which were subsequently incubated at 4°C for 2 h and then left to rest at room temperature for 20 min to allow zoospore release.

### Effects of Single Strains vs. Strain Combinations on Disease Protection in a Leaf Disk Assay

The 3rd and 4th leaves of greenhouse grown potato plants of the cultivars Bintje, Lady Claire and Victoria were harvested 7 weeks after emergence. Using a cork borer, leaf disks (1.8 cm diameter) were cut and positioned abaxial face up on 1% water agar plates. Droplets of 10 μL of a mixture of bacterial and sporangial suspensions were pipetted in the center of each leaf disk, at final concentrations of 125,000 sporangia/mL for *P. infestans* and of OD_570_ = 0.9 for single bacterial strains (simple), OD_570_ = 0.45 for combinations of two strains (double), and OD_570_ = 0.3 for combinations of three strains (triple). For negative control plates, bacterial suspensions were replaced with a 0.9% NaCl solution. The plates were stored at 18°C (the ideal growth temperature for *P. infestans*) and at high humidity in the dark. After seven days, the plates were photographed. Severity of infection was assessed through estimation of sporangiophore development using a macro-instruction in ImageJ as described previously (Guyer et al., 2015). Per experiment, each treatment was tested in five replicates consisting of five disks from five different plants in Lady Claire and Victoria, and in ten replicates consisting of ten leaf disks from ten different plants in Bintje (because we had more plants available for Bintje than for Lady Claire and Victoria). Each batch of five plants could be used to assess the efficiency of 27 different treatments by comparing infection severity in treated vs. untreated disks coming from the same plants. To enable comparison between different batches of plants, the infection severity quantified on untreated control disks was set to 100% and the infection severity of treated disks was expressed as percentage of the control (relative infection severity). Finally, treatment efficiency was calculated with the following formula: Treatment efficiency (%) = 100 – (relative infection severity of treatment). With this calculation, a treatment efficiency of 100% corresponds to no infection, an efficiency of 0% corresponds to the same infection as in untreated controls and a negative value indicates higher infection severity in treated disks compared to untreated controls.

### Effects of Strain Combinations on *P. infestans* Mycelial Growth

Bacterial strains and *P. infestans* were co-inoculated on Pea agar plates. Three drops of 10 μL of bacterial suspensions (prepared as described above) were pipetted on the medium equidistantly and 10 mm from the border of the plates. One 5 mm plug of a 14-day-old *P. infestans* culture was placed in the center. Negative control plates contained 10 μL of NaCl 0.9% instead of bacterial cell suspension. The plates were prepared in three replicates and were incubated in the dark for 6 days at 18°C before being photographed. Mycelium growth was assessed by measuring the growth area of *P. infestans* using the ImageJ software. Relative mycelial growth was calculated by dividing the mycelial area quantified in treated plates by the mycelial area quantified in negative control plates (in the absence of bacteria). Treatment efficiency was then calculated as above (100 – relative mycelial growth).

### Effects of Strain Combinations on *P. infestans* Zoospore Release

Cold shock was applied to sporangia suspensions freshly mixed with bacterial suspensions. Thirty μL of the mixture were pipetted onto a 24-well plate (Costar), which contained one well per treatment. Per well, one picture was taken at 4-fold magnification using a Cytation5 plate reader (Biotek, United States) once the zoospores had settled and once more after shaking the plate to insure even distribution of the zoospores for counting. The experiment was repeated three times. An average of the three replicate experiments was calculated for each treatment. Relative zoospore release was calculated by dividing the number of zoospores released in treated samples by the average number of zoospores released in the untreated samples (negative controls non-exposed to bacteria). Treatment efficiency was calculated as above (100 – relative zoospore release).

### Growth/Survival of Bacteria as Single Strains vs. in Combinations With Other Strains

To find out whether the development of single bacterial strains was affected by the presence of other strains, the growth/survival of each bacterial strain was assessed alone and in combination. Rifampicin resistant and wild type bacteria were mixed in equal densities in NaCl (0.45%) and incubated at 18°C without shaking, to mimic the conditions of the leaf disk experiment. We used 0.45% NaCl to have the same concentration as in leaf disk experiments, where bacterial cells suspended in 0.9% NaCl were mixed with equal volumes of *P. infestans* sporangia suspended in water. After 1 day and after 5 days, 10 μL of bacterial suspension were 10-fold serially diluted in NaCl (0.9%) and 15 μL of the diluted suspensions were plated onto differentially selective media to discriminate the two or three different strains, taking advantage of rifampicin resistance vs. sensitivity, of ability vs. inability to grow on PIA and of different colony morphology. Colony forming units (CFUs) were counted after two or three days depending on the medium.

### Statistical Analyses

All statistical analyses were performed using R software (Sasaki et al., 2005). When possible, one-way or two-way ANOVA was performed followed by Dunnett’s test or Tukey’s HSD test using agricolae and multcomp packages. If needed, boxcox transformation computed via the MASS package was used to meet normality and homogeneity of variances. For effects on zoospore release and mycelial growth data, Kruskal–Wallis test was used to discriminate between treatments.

## Results

### Disease-Inhibiting Effects of Nine *Pseudomonas* Strains in Single, Dual and Triple Combinations

**FIGURE 1:**
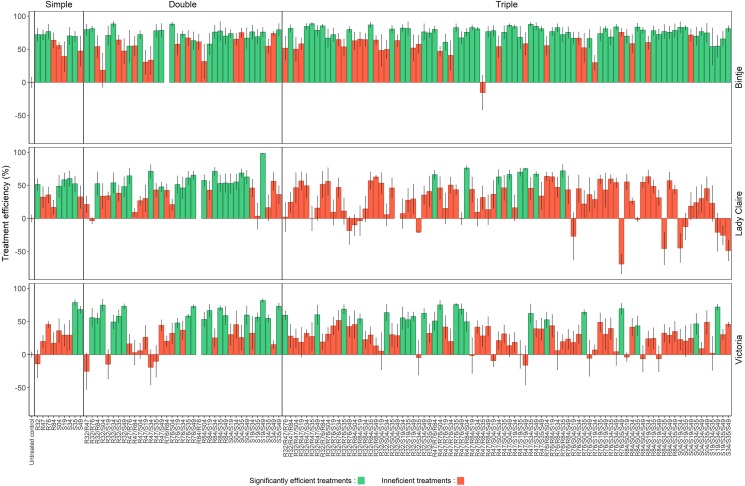
*Phytophthora infestans* relative infection severity in leaf disks of three potato cultivars treated with nine *Pseudomonas* applied as single strains vs. in double or triple combinations. Results are expressed as treatment efficiency (100 – relative infection severity in each treatment compared with the untreated leaf disks), with means and standard errors of 10 replicates for Bintje and five replicates for Lady Claire and Victoria (see Materials and Methods for more details). Two-way ANOVA revealed interactions between cultivar and treatment effects, resulting in separate analysis of all three cultivars. Significantly efficient treatments (leading to significant infection reductions compared with the untreated control) are marked in green (Dunnett’s test, *p* < 0.05). Inefficient treatments are marked in red (Dunnett’s test, *p* > 0.05).

Nine *Pseudomonas* strains previously isolated from the rhizosphere (R) or phyllosphere (S) of field-grown potatoes and displaying various levels of *Phytophthora*-inhibiting activity *in vitro* were selected for this experiment. To determine whether these strains would confer higher protection when applied in combinations than when applied as single strains, we carried out a leaf disk infection experiment with 129 treatments (9 single strains, their 36 dual and 84 triple combination possibilities) using three potato cultivars differing in late blight sensitivity. We selected Bintje as highly sensitive, Lady Claire as sensitive and Victoria as moderately tolerant. This experiment revealed a strong cultivar effect, with higher overall protection efficacy in Bintje than in Lady Claire and Victoria (Figure 1). Among the nine treatments with single strains, only one strain (S35) significantly reduced disease progression in all three varieties, while 7 offered protection on some but not all varieties and one (R84) was inefficient in all varieties. Among the dual combinations, six (out of 36 possible combinations) provided protection on all three cultivars, i.e., R32/S34, R76/S49, R84/S35, R84/S49, S04/S49, and S19/S49. This latter dual combination offered best protection in terms of quantitative disease inhibition (Figure 1). Interestingly, four out of six of these efficient combinations contained the strain S49, while only one contained the strain S35, which showed consistent protection when applied as single strain. Among the 84 possible triple combinations, only seven were able to significantly reduce disease progression in Lady Claire, among which two were also efficient on the two other varieties. When considering less stringent conditions, e.g., triple combinations able to reduce disease progression in at least two of the three varieties, 16 combinations were found to be efficient, among which seven contained strain S35 and six contained strain S49, indicating putative synergistic effects of these two strains when applied in combinations with two additional other strains. In addition to the overrepresentation of S35 and S49 in the efficient combinations, we noticed that the duo R47/S35 combined with either S19, S34, or R76, yielded significant protection against *P. infestans* infection.

**FIGURE 2:**
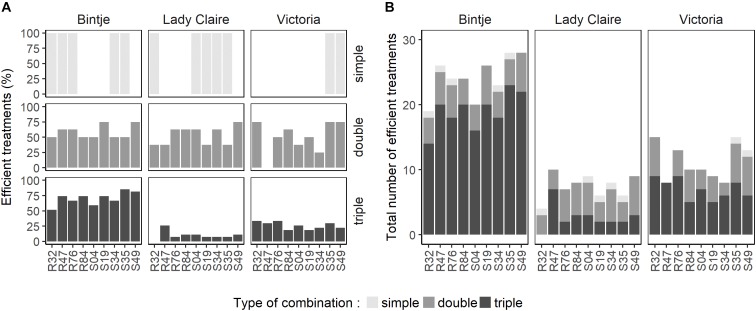
Evaluation of combinatory competence of individual *Pseudomonas* strains when enlisted in single, double or triple combination treatments. For each *Pseudomonas* strain, all efficient treatments featuring the respective strain were counted per cultivar. Treatments were considered efficient if they significantly reduced sporangiophore development on leaf disks (based on data from Figure 1). **(A)** Percentage of efficient treatments obtained per bacteria per treatment type (8 possible treatments per strain in double and 26 possible treatments in triple combinations). **(B)** Overall number of efficient treatments per bacteria in single use or in combination with one or two other strains.

To gain a general view on the performance of the strains in single, dual and triple combinations, we calculated the percentage of efficient treatments, i.e., those significantly reducing disease symptoms, in the three varieties and for each strain in its respective modes of application (single or combinations) (Figure 2A). The relative efficiency of the strains in the different modes of application strongly depended on the variety, yet some strains showed consistent differences between the three application modes: R84 was consistently more efficient when applied with one or two other strains than when applied alone. The same was observed for S49 on Bintje and Lady Claire. In Bintje, the strains performed generally better in triple combinations than in dual combinations in terms of percentage of efficient treatments, while the opposite trend was observed in Lady Claire and Victoria (Figure 2A). Because the total bacterial cell density was not the same in single, dual and triple treatments (OD of 0.9 for single, 0.45 for dual and 0.3 for triple treatments), we wondered whether the low percentage of efficient treatments in triple combinations on Lady Claire and Victoria could be explained by these differences in inoculum density. We therefore tested the protective effect of selected single strains applied in the three different densities. This revealed that in Lady Claire and Victoria, cell density in the range of OD = 0.3 – 0.9 did generally not influence the extent of protection conferred by the strains (Supplementary Figure S1). However, in Bintje, a dose-dependent protective effect was observed, with applications at OD = 0.3 generally being less efficient than applications at OD = 0.9, although both concentrations were able to significantly reduce disease symptoms (with the exception of strains R32 and S19) (Supplementary Figure S1). Therefore, the high percentage of efficient treatments in triple combinations in Bintje that were observed despite overall lower cell density were likely due to synergetic effects between the strains, that were able to compensate the overall lower cell density.

To better understand the mechanisms underlying disease inhibition by strain combinations, we selected a subset of five strains to compare their effect as single strains and as double/triple combinations on two main developmental stages of *P. infestans*, mycelial growth and zoospore release. The selection of these five strains was carried out such as to maximize the chance to see synergetic effects and was based on the number of efficient treatments per strain (Figure 2B). We therefore selected R47, S19, S35 and S49 based on the results on Bintje, and included R32 for its efficient protection of Victoria (Figure 2B). R32 was remarkably efficient in protecting Victoria when applied in dual combinations, where it led to significant disease reduction when combined with six of the eight other strains (Figure 1). The last criterion for strain selection was to ensure some diversity, both in terms of phylogeny and in terms of origin of isolation (rhizosphere vs. phyllosphere). The main properties of these five selected strains are listed in Table 1.

**Table 1:** Properties of the five selected *Pseudomonas* strains.

Strain	Origin of isolation	Phylogeny	HCN	Phenazines
R32	Rhizosphere	*P. putida*	Yes	No
R47	Rhizosphere	*P. chlororaphis*	Yes	Yes
S19	Phyllosphere	*P. frederiksbergensis*	No	No
S35	Phyllosphere	*P. fluorescens*	No	No
S49	Phyllosphere	*P. fluorescens*	Yes	No

*Phylogenetic analysis was based on (De Vrieze et al., 2015; Hunziker et al., 2015). HCN and Phenazines are listed as the two major known determinants of anti-Phytophthora activity (Hunziker et al., 2015; Morrison et al., 2016).*

### Mycelial Growth Inhibition by Five Selected Strains and Their Dual and Triple Combinations

**FIGURE 3:**
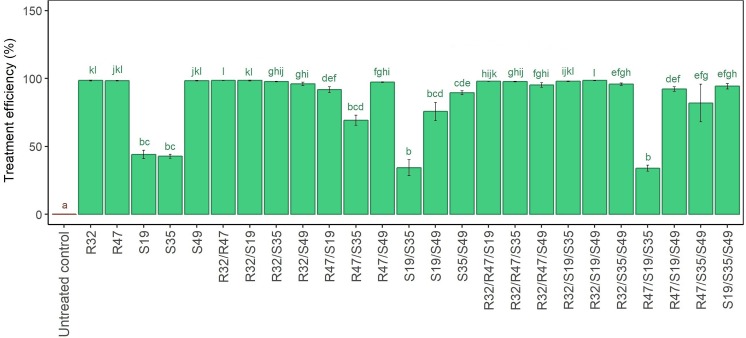
Relative mycelial growth of *P. infestans* when exposed to single, double and triple combinations of five *Pseudomonas* strains in a dual culture Petri dish assay. Untreated controls represent *P. infestans* grown without bacteria. Relative mycelial growth was calculated by dividing the mycelial area obtained in the respective treatments with that obtained in the untreated controls (not exposed to bacteria). Results are means of three replicates from the same experiment. They are expressed as treatment efficiency and calculated as above (100 – relative mycelial growth). Letters indicate significant differences between treatments according to Kruskal–Wallis test (*p* < 0.05, *n* = 3).

When inoculated alone, three strains (R32, R47 and S49) were able to inhibit fully the mycelial growth of *P. infestans*, while the two others (S19 and S35) induced more moderate, but still significant growth inhibition. The dual combination of the two “weaker” strains did not result in stronger mycelial growth reduction, nor did the “stronger” strains lose their activity when mixed with other strong strains (Figure 3). Interestingly, when either S19 or S35 were mixed with R32, the same complete inhibition of mycelial growth was observed as when R32 was inoculated alone. However, this was not the case with either R47 or S49, whose effects were weakened when mixed with S19 or S35, especially in the S49/S19 and in the R47/S35 combinations, and to a lesser extent in the S35/S49 combination. Concerning the triple combinations, the beneficial impact of R32 was also clearly visible, since its addition conferred strong activity to any couple of strains, including the inactive S19/S35 but also the moderately active R47/S35 and S19/S49 (Figure 3). In contrast, adding the active R47 to the “inactive” couple S19/S35 did not improve mycelial inhibition efficiency. From this experiment, it appeared that (i) R32 was the best helper in dual and triple combinations, (ii) S49 only improved the efficiency of S19/S35 but had no positive effect on the other dual combinations, and (iii) R47 was not able to increase the efficiency of S19/S35 and had generally little positive influence in triple combinations (Figure 3). Here as well as in the leaf disk experiments, it should be noted that single strains were applied with an optical density of 0.9, against 0.45 for the double and 0.3 for the triple combinations. We therefore tested whether cell density changes at the start of the experiment would influence the extent of mycelial growth inhibition and this was not the case for any of the strains (Supplementary Figure S2).

### Zoospore Release Affected by Five Selected Strains and Their Dual and Triple Combinations

**FIGURE 4:**
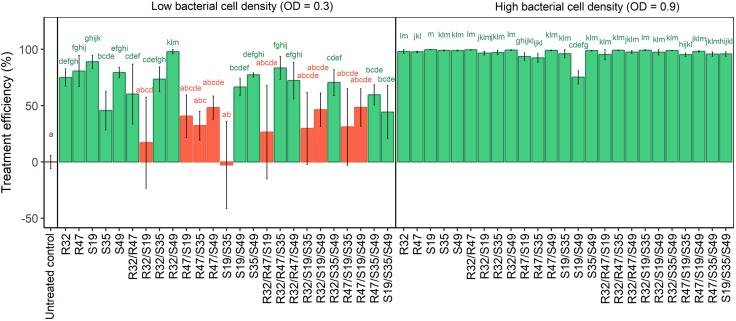
Relative zoospore release from *P. infestans* sporangia exposed to single, double and triple combinations of five *Pseudomonas* strains applied at two different cell densities. Sporangia suspensions pre-mixed with saline instead of bacterial cell solution were used as untreated controls. Released zoospores were counted and the relative release rate was calculated by dividing values obtained for treatments by those obtained for the untreated controls. Results are means of three experiments with one sample per treatment. They are expressed as treatment efficiency and calculated as above (100 – relative zoospore release). Letters indicate significant differences between treatments according to Kruskal–Wallis test (*p* < 0.05, *n* = 3).

Preliminary experiments revealed that the total cell density of the strains in single or mixed applications influenced their effect on *P. infestans* zoospores (Supplementary Figure S3), therefore this assay was carried out with two different optical densities, a high one (OD = 0.9) and a lower one (OD = 0.3). When bacteria were applied at an OD of 0.9, all treatments significantly and drastically inhibited zoospore release (Figure 4). Interestingly, the dual combination of S19/S49 caused lesser reduction in zoospore release than each of the strains applied individually, although it was still significant compared with the control. This decrease in activity was also observed in the more diluted applications (OD = 0.3) and was even more pronounced when S19 was mixed with R32, R47 or S35, where it led to complete loss of activity. Adding any other strain to the couples S19/R32, S19/R47 or S19/S35 could not restore the significant activity observed with the strains applied alone (Figure 4). In contrast to the observed antagonistic effects between S19 and other strains, some synergetic effects could also be seen in the experiment carried out with lower cell density: mixing S49 with R32 resulted in almost total inhibition of zoospore release, while the single strains still allowed ca. 20% of the sporangia to release the zoospores. Likewise, mixing S49 with S35 resulted in more consistent (less variable) inhibition of zoospore release than either of the strains applied alone. Remarkably, all combinations of strains containing this couple (S35/S49) significantly reduced zoospore release, which was not the case for any other couple. When comparing the total number of efficient combinatory treatments for each strain (out of 10 possible dual and triple combinations), we observed that S49 scored best (7/10), followed by S35 and R32 (6/10), while combinations containing R47 (4/10) and especially S19 (2/10) were much less efficient. This suggests that in the experimental setup used to assess zoospore release, S49, S35 and R32 had a beneficial effect on other strains present in the respective mixtures, while R47 and especially S19 had deleterious effects on the same strains (Figure 4).

### Survival and Growth of Five Selected Strains Alone and in Their Dual and Triple Combinations

**FIGURE 5:**
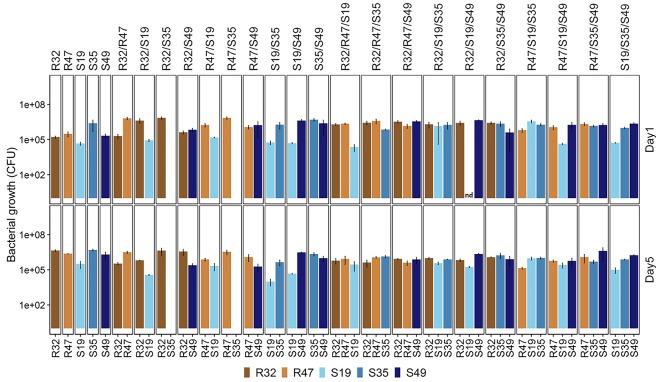
Abundance of each bacterial strain (alone and in dual vs. triple combinations) after 1 **(top)** and 5 **(bottom)** days of incubation in NaCl (0.45%). Results are means of three replicates from the same experiment. nd, no data (this strain’s CFUs could not be counted due to a technical problem but the strain was present, as indicated by its abundance after 5 days). Please refer to Table 2 for statistical analysis of the data after 5 days.

Some of the results described above hinted at possible direct stimulating or inhibiting effects of strains on each other. To assess whether co-incubation in conditions similar to those applied in the leaf disk assay would lead to preferential survival/growth of specific strains, we incubated the five selected strains alone, as well as in dual and triple combinations for five days in physiological solution and quantified their relative abundance after one day and at the end of the experiment. For this experiment, all treatments had the same global cell density at the start of the experiment (as estimated by optical density), meaning that each individual strain started with half the inoculum vs. 1/3 of the inoculum in dual vs. triple combinations compared with the treatment where it was inoculated alone. Despite these differences, the total CFU counts (taking all strains together) at the end of the experiment were much higher for dual and triple combinations compared to single inoculations (Figure 5), indicating that (i) strains were able to compensate the lower initial cell density over the 5 days of growth, and (ii) strains generally did not grow at the expense of each other, although they were incubated in saline solution only (no nutrient supply). When inoculated alone, four of the five strains developed to a density of roughly a million CFU/mL after 5 days, while their abundance was slightly lower after 1 day, indicating mild growth from day 1 to day 5 even in these nutrient poor conditions. S19 was already less abundant than the others after 1 day, and this strain hardly grew or even decreased in abundance depending on the combinations during the four following days (Figure 5 and Table 2). Overall, being incubated with different partners did not affect all strains in the same way (Table 2): S35 and, to a lesser extent, R32, were inhibited in their growth in many of the dual and triple combinations compared to when they were inoculated alone, while R47 grew less well mainly in triple combinations but was not affected by dual combinations. S19 was only growing less well in combinations than alone in three out of 10 possible combinations and S49 was only affected by the presence of R47 in dual combination, but otherwise grew as well with any partner as alone (Table 2 and Figure 5). A striking fact was observed in the case of S35: beyond its general decrease in abundance when mixed with other strains, it appeared to be completely outcompeted or even killed when incubated with either of the two rhizosphere strains R32 and R47. However, these latter strains did not seem to profit from the presence of S35, since their abundance was not significantly higher in the presence of S35 than in its absence (Figure 5 and Table 2). This inhibition of S35 in presence of either R32 or R47 was rescued when any additional strain was present, since S35 grew normally in all tripartite combinations tested, even in the combination with R32 and R47.

**Table 2:** Statistical analysis of the abundance of the five selected strains alone or in dual vs. triple combinations.

R32			R47			S19			S35			S49		
**R32**	**30.0**	**a**	**R32/R47**	**28.7**	**a**	**R47/S19/S35**	**30.3**	**a**	**S35**	**32.0**	**a**	**S19/S49**	**30.0**	**a**
R32/S49	28.3	a	R47/S35	27.8	ab	R32/S19/S35	24.8	ab	S35/S49	26.3	ab	R32/S19/S49	25.7	ab
R32/S35	27.0	ab	R47	26.0	abc	S19	22.2	ab	R32/R47/S35	23.7	abc	S19/S35/S49	23.0	abc
R32/S35/S49	22.0	abc	R32/R47/S35	19.8	abcd	R32/S19/S49	20.2	abc	**R32/S35/S49**	**21.7**	**bc**	R47/S35/S49	21.3	abcd
**R32/S19/S35**	**18.5**	**bcd**	R47/S49	17.0	bcd	R47/S19	19.7	abc	**R47/S19/S35**	**20.0**	**bc**	S49	20.0	abcde
**R32/R47/S49**	**15.7**	**cde**	R47/S19	15.7	cd	R32/R47/S19	17.8	bcd	**R32/S19/S35**	**17.0**	**cd**	S35/S49	15.7	bcdef
**R32/S19/S49**	**11.7**	**de f**	R47/S35/S49	15.5	cd	R47/S19/S49	17.5	bcd	**S19/S35/S49**	**16.8**	**cd**	R32/R47/S49	14.7	bcdef
**R32/S19**	**11.2**	**de f**	**R32/R47/S19**	**12.3**	**de**	S19/S35/S49	14.8	bcd	**R47/S35/S49**	**11.3**	**de**	R32/S35/S49	12.5	cdef
**R32/R47/S19**	**11.0**	**de f**	**R47/S19/S49**	**12.3**	**de**	**S19/S49**	**10.3**	**cde**	**S19/S35**	**10.2**	**de**	R47/S19/S49	9.8	def
**R32/R47/S35**	**7.3**	**ef**	**R32/R47/S49**	**9.0**	**de**	**R32/S19**	**7.2**	**de**	**R32/S35**	**4.0**	**e**	R32/S49	8.5	ef
**R32/R47**	**4.3**	**f**	**R47/S19/S35**	**2.8**	**e**	**S19/S35**	**2.2**	**e**	**R47/S35**	**4.0**	**e**	**R47/S49**	**5.8**	**f**

*Treatments are ordered from highest (top) to lowest (bottom) abundance. For each strain (column), different letters indicate statistically different values of the treatments according to Kruskal–Wallis test, *p* < 0.05, n = 3. Bold font indicates significantly lower abundance of the combined treatments compared with the control (single strain).*

## Discussion

Efficient control of late blight by bacterial biocontrol agents has been observed in few cases in greenhouse or even field experiments (Puopolo et al., 2014; Caulier et al., 2018), but most of the studies reported lack of reproducibility in protection against this disease (reviewed in Dorn et al., 2007; Axel et al., 2012). Indeed, in contrast to a synthetic molecule acting directly on a specific target of the pathogen, biocontrol agents that are applied, e.g., on leaves need (i) to efficiently compete with the native microbiota to colonize this environment, and (ii) to survive there despite exposure to UV and to rapidly changing temperature and humidity. Once established, they can produce bioactive molecules that either trigger the host plant’s immune defense or that directly inhibit the pathogen’s development. *Phytophthora infestans*, as many other plant pathogens, undergoes different developmental stages during the infection season, such as producing/releasing spores (sporangia and zoospores) or growing mycelium to colonize the host tissues (Fry, 2008). Ideally, control measures should target as many of these stages as possible to maximize efficiency.

One possible way to increase the chances for biocontrol agents to overcome the above-mentioned hurdles consists in using mixtures of strains rather than single agents, to increase both functional polyvalence (targeting different stages of the pathogen life cycle) and redundancy (maximizing the chances of successful host plant colonization in various environmental conditions). This “polymicrobial” approach has drawn considerable attention in recent years, although most studies have so-far focused on mixing well-known, commercially available microbial agents such as *Trichoderma* and *Bacillus/Pseudomonas* or mycorrhizal fungi and nitrogen-fixing bacteria (Xu et al., 2011; Reddy and Saravanan, 2013; Sarma et al., 2015; Parnell et al., 2016). Few studies compared the effect of such strain combinations with that of the respective strains applied alone, and they came to divergent conclusions: Pertot et al. (2017) performed a 4-year field study on biological control of *Botrytis cinerea* in grapevine using a combination of two fungi (*Trichoderma*, *Aureobasidium*) and a *Bacillus*. They observed good efficacy for each of the antagonist but no additive value of combining the three (Pertot et al., 2017). Using five commercially available biocontrol agents (two based on *Bacillus*, one on *Streptomyces* and two on *Trichoderma* strains) against *Phytophthora ramorum* in a detached leaf assay, Elliott et al. (2009) observed lower efficacy of the mixture compared to some of its individual components, suggesting antagonistic effects between the different strains composing the mixture (Xu et al., 2011). The performance of strain combinations compared to individual strains might also depend on the targeted disease, as observed in rice for a dual treatment of *Trichoderma* and *Pseudomonas* strains, which was more effective than its single constituents against blast (caused by a fungus) but not against blight (caused by a bacterium) (Jambhulkar et al., 2018). In contrast, protection against *Ralstonia*-induced wilt in tomato was much higher when a mixture of eight *Pseudomonas* strains was applied than when the strains were applied individually (Hu et al., 2016).

Most of the above-mentioned studies used strains available as commercial products or in strain collections; however, these strains might not be adapted to the plant host and its pathogens, depending on their origin of isolation. Moreover, most screening efforts leading to the discovery (and putative registration) of antagonist strains have been done in *in vitro* experiments, which does not necessarily reflect the true antagonistic potential *in planta* or even in field conditions. In the present study, we investigated whether protective effects of *Pseudomonas* strains would be higher when applied in combinations than as single strains. Using nine potato-associated *Pseudomonas* strains, we performed a leaf disk infection assay with all 129 possible dual and triple combinations to circumvent the bias of the *in vitro* selection procedure. We performed this leaf disk screening on three different potato cultivars since we expected that the strain performance would vary according to the host plant genotype and sensitivity to late blight. As expected, a strong cultivar effect was observed, but surprisingly, best overall protection occurred on Bintje, which is most sensitive to late blight, while the two other cultivars were less efficiently protected by the strains (Figure 2B). This, however, might be at least partially because the screening on Bintje was carried out on ten leaf disks, while only five leaf disks per treatment were analyzed for the other two cultivars (see Material and Methods for the underlying reason). Only one strain, *P. fluorescens* S35, conferred significant protection on all three cultivars when applied alone, but this strain was less represented among efficient dual combinations than, e.g., *P. fluorescens* S49. This might be due to inability of S35 to compete with other *Pseudomonas* strains, as evidenced by the fact that in most combinations tested, S35 grew less well than when incubated alone (Table 2). In contrast, S49 could grow to the same level when co-incubated with any other strain we tested, except with R47, where it was slightly inhibited in its growth. Interestingly, when S35 was co-incubated with either R32 or R47, it could not be recovered after five days, suggesting strong inhibition or even killing of S35 by R32 and R47. This observation could explain the loss of activity of S35 when mixed with R47 on Lady Claire and Victoria, on which R47 was not active on its own, while R47/S35 was still active on Bintje, where R47 was active on its own (Figure 1). Likewise, S49, which was offering significant protection on Victoria when applied alone, lost its activity when mixed with R47, while it kept it when mixed with other strains that did not interfere with its growth. These results indicate that the mutual influence of strains on each other, when incubated in very low nutrient conditions, might be a useful parameter to investigate when designing microbial consortia for protection against diseases.

**FIGURE 6:**
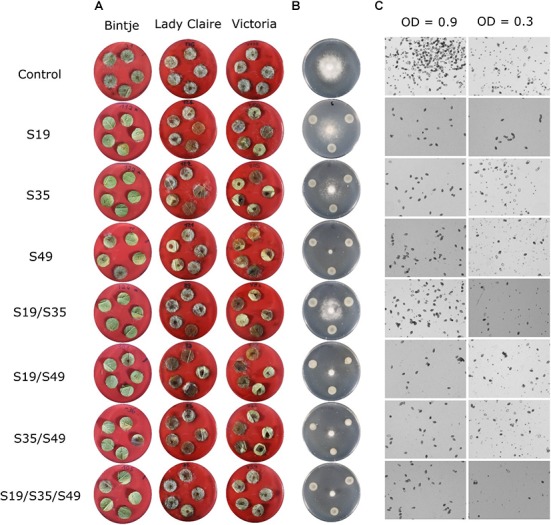
Representative pictures of the effects of S19, S35, and S49 applied as single strains or in dual vs. triple combinations. **(A)** Leaf disk assay on the three cultivars; **(B)** mycelial growth; **(C)** zoospore release with total bacterial cell densities of 0.9 (left) and 0.3 (right). Please see Materials and Methods for details.

The screening of the 129 different treatments did not lead to an overall “champion” combination, but it highlighted the consistent protective activity of some strains, either when applied alone (S35), or in dual combinations (S19/S49). This latter combination was particularly interesting since it was efficient on all three cultivars, but when applied alone, neither strain was efficient on Bintje, only S19 was efficient on Lady Claire and only S49 was efficient on Victoria, thereby suggesting a synergetic effect between the two strains. Likewise, the dual combination S35/S49 was efficient on Bintje although only S35 was efficient on this cultivar when applied alone (Figures 1, 6A). We wondered whether such synergetic effects could be due to differential modes of action of the different strains, e.g., inhibiting specifically the mycelial or spore stage of the pathogen, and tested these three potato phyllosphere isolates, together with two rhizosphere isolates previously shown to display strong anti-*Phytophthora* activity *in vitro* (Guyer et al., 2015; Hunziker et al., 2015).

We observed that the dual combination of S19/S49, which was particularly efficient on leaf disks, was only moderately inhibiting the mycelial growth of *P. infestans* in the *in vitro* assay, much less than when S49 was applied alone. However, all triple combinations containing S19/S49 (including that with the otherwise moderately active S35) were highly efficient in inhibiting mycelial growth, in contrast to those containing R47/S35 (Figure 3). In the natural leaf infection cycle, as well as in our leaf disk assays, the infection starts with a sporangium that, depending on temperature, can either directly germinate or release motile zoospores (Fry, 2008). Originally we aimed at investigating the effects of the five selected strains on these two processes but due to unknown reasons and despite repeated trials in different conditions, the harvested *P. infestans* sporangia did not germinate (even in the control) as they did previously in our hands, but consistently released zoospores. We therefore focused on analyzing how the five selected strains would affect this important route of infection in single, dual and triple combinations. Interestingly, the strain inhibiting this stage of *P. infestans* development in the strongest and most consistent way was S19, while S35 was the least active one (Figure 4). However, mixing S19 with any other strain but S49 led to loss of the activity. Among triple combinations, all those containing both S35 and S49 led to significant inhibition of zoospore release, suggesting good tolerance of these strains toward additional members of the tripartite consortium.

In summary, our screening of 129 different treatments of single, dual and triple strain combinations against *P. infestans* on three potato cultivars led to the observation that despite strong cultivar specificity, some strains showed strong and consistent protective effects, either when applied alone (S35) or in combination (S19/S49). The effects of these three phyllosphere strains on disease development, mycelial growth and zoospore release are shown through representative picture of the respective assays in Figure 6. When investigating the effect of these strains on each other’s growth, we observed that S35 was less able to compete with other strains than S49 or even S19 (Figure 5 and Table 2), possible explaining its better leaf disk performance when applied alone than in combination. The successful combination of S19 and S49 could be explained by their different mode of action: while S49 had much stronger inhibiting effect on mycelial growth than S19, S19 was a very efficient inhibitor of zoospore release. Despite their difference, S19 and S49 were able to maintain sufficient population densities when grown together (Figure 5), which is a prerequisite for synergetic effects.

In previous studies, we had considered S19, S35 and even S49 as among the lesser active strains, because our activity screening was performed mostly on *in vitro* tests assessing mycelial growth inhibition in dual assays (Guyer et al., 2015; Hunziker et al., 2015). Interestingly, when screening for protection using leaf disks rather than *in vitro* assays, these three phyllosphere strains turned out to be the most promising ones, which might be due to a particular ability to survive on leaf tissues or to cope with plant defenses. In dual and triple combinations, mixing of either S19/S49 (leaf disks) or S35/S49 (zoospore release assay) proved efficient in inhibiting *P. infestans* development. This good compatibility of strains sharing the same – phyllosphere – origin was not observed when mixing strains from the phyllosphere with strains from the rhizosphere, which might indicate that these strains have different requirements with respect to environmental conditions. In addition to leaf blight, *P. infestans* also causes tuber blight and it would be interesting to see whether rhizosphere isolates would prove more efficient than phyllosphere isolates for this particular form of the disease. In contrast to foliar blight, tuber blight was shown in an earlier study to be efficiently controlled by a mixture of four strains, among which three were fluorescent pseudomonads (Slininger et al., 2007). These strains were originally isolated from suppressive soils supplemented with tuber slices (Schisler and Slininger, 1994) and their protection efficacy was much higher in the mixture than with either of the strains applied alone, which highlights the potential of such host plant- or even host-tissue derived consortia to fight oomycete diseases.

Overall, our study clearly shows the potential added value of combining different, but compatible strains, with the example of a dual combination that led to stronger and more consistent protection than that obtained with the single strains. This study also highlights the complexity of interactions taking place even in such limited tripartite consortia. When increasing the number of partners, much higher complexity shall be expected, opening a wide range of fascinating questions related to the role of each strain in the consortium and the broader community (Lindemann et al., 2016), as recently exemplified by Niu and co-workers, with the identification of one “keystone” species in a 8-member consortium (Niu et al., 2017). Beyond the traditional way of systematically isolating strains to test them later in single or combined applications, future endeavors might rely on the plant’s ability to specifically recruit beneficial microbes when facing a particular pathogen attack, as recently demonstrated in the model plant *Arabidopsis thaliana* (Berendsen et al., 2018). Such recruited microbes might then be assembled in synthetic communities and investigated for protective potential against the original pathogen, as well as for other desired features to be conferred to the plant. This new and booming field of microbiome management is likely to provide innovative alternatives to our current ways of protecting plants against diseases (Herrera Paredes et al., 2018; Syed Ab Rahman et al., 2018), which would ideally be combined with more traditional strategies such as adapted crop management or selection of resistant varieties to achieve more durable and sustainable crop protection.

## Author Contributions

LW and MDV designed the research. NV, FG, and MDV performed the experiments. MDV analyzed the data. LW and MDV wrote the manuscript with help from FG and NV.

## Conflict of Interest Statement

The authors declare that the research was conducted in the absence of any commercial or financial relationships that could be construed as a potential conflict of interest.

## Supplementary Material

FIGURE S1 *Phytophthora infestans* relative infection severity in leaf disks of three potato cultivars treated with five *Pseudomonas* applied as single strains at low, medium and high cell density (OD_570_ = 0.3, OD_570_ = 0.45, OD_570_ = 0.9). Results are expressed as treatment efficiency (100 – relative infection severity in each treatment compared with the untreated leaf disks), with means and standard errors of 15 replicates for Bintje, Lady Claire and Victoria. Within each variety, different letters indicate significant differences between the treatments according to Tukey’s HSD test (*P* < 0.05, *n* = 15).

FIGURE S2 Relative mycelial growth of *P. infestans* when exposed to five *Pseudomonas* applied as single strains at three different bacterial cell densities in a dual culture Petri dish assay. Untreated controls represent *P. infestans* grown without bacteria (see Materials and Methods for details). Relative mycelial growth was calculated by dividing the mycelial area obtained in the respective treatments with that obtained in the untreated controls (not exposed to bacteria). Results are means of four replicates from the same experiment. They are expressed as treatment efficiency and calculated as above (100 – relative mycelial growth). Letters indicate significant differences between treatments according to Kruskal–Wallis test (*p* < 0.05, *n* = 4).

FIGURE S3 Relative zoospore release from *P. infestans* sporangia exposed to five *Pseudomonas* strains applied at three different cell densities. Sporangia suspensions pre-mixed with saline instead of bacterial cell solution were used as untreated controls. Released zoospores were counted and the relative release rate was calculated by dividing values obtained for treatments by those obtained for the untreated controls (see Materials and Methods for details). Results are means of three experiments with one sample per treatment. They are expressed as treatment efficiency and calculated as above (100 – relative zoospore release). Letters indicate significant differences between treatments according to Kruskal–Wallis test (*p* < 0.05, *n* = 3).
