# Supplementary figures and images for: Combining Different Potato-Associated Pseudomonas Strains for Improved Biocontrol of Phytophthora infestans
Source: Front Microbiol. 2018 Oct 29;9:2573. doi: 10.3389/fmicb.2018.02573 (PMC6215842; doi:10.3389/fmicb.2018.02573)

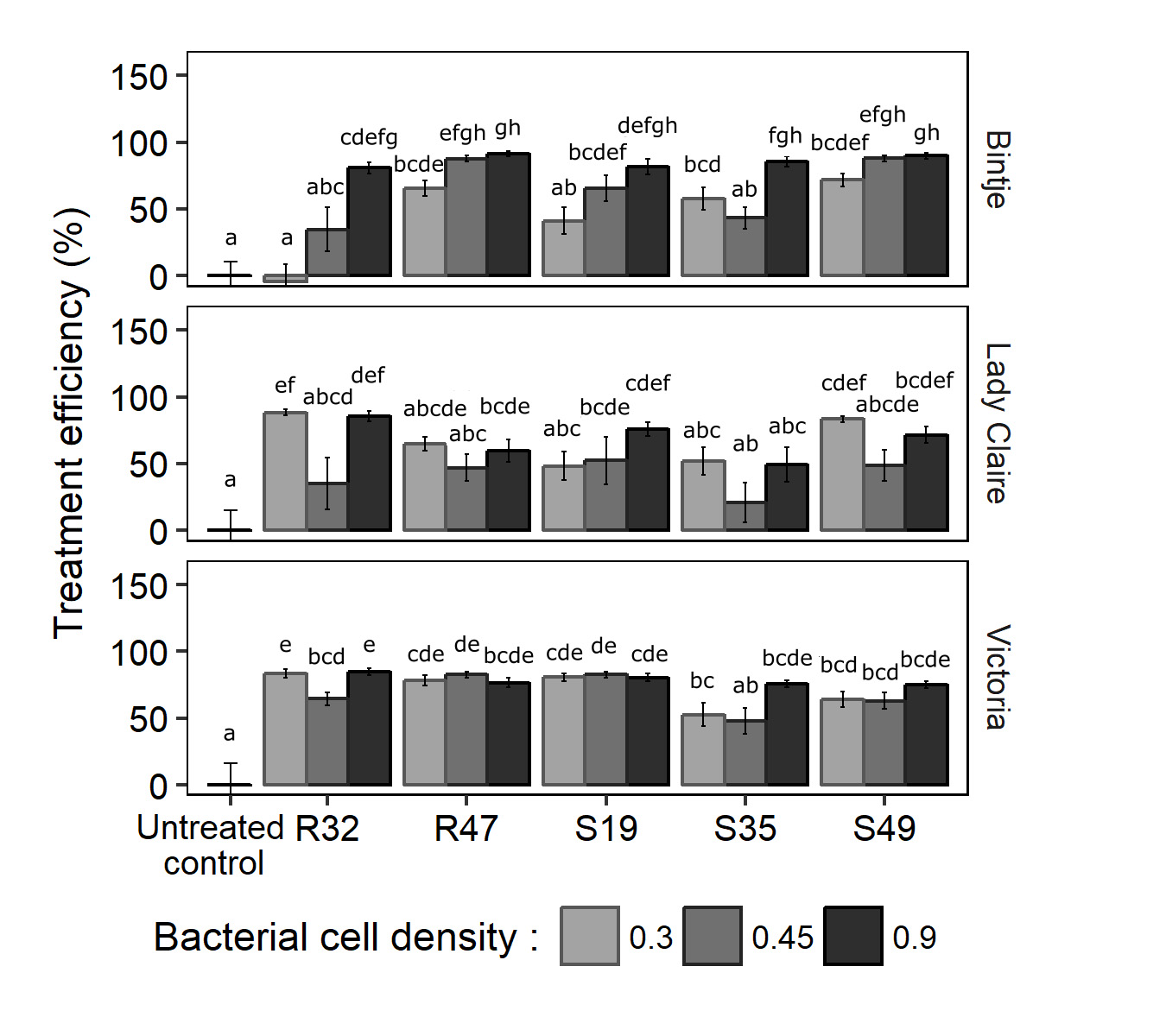

Supplement: FIGURE S1 — Phytophthora infestans relative infection severity in leaf disks of three potato cultivars treated with five Pseudomonas applied as single strains at low, medium and high cell density (OD570 = 0.3, OD570 = 0.45, OD570 = 0.9). Results are expressed as treatment efficiency (100 – relative infection severity in each treatment compared with the untreated leaf disks), with means and standard errors of 15 replicates for Bintje, Lady Claire and Victoria. Within each variety, different letters indicate significant differences between the treatments according to Tukey’s HSD test (P < 0.05, n = 15). [file Image_1.JPEG]

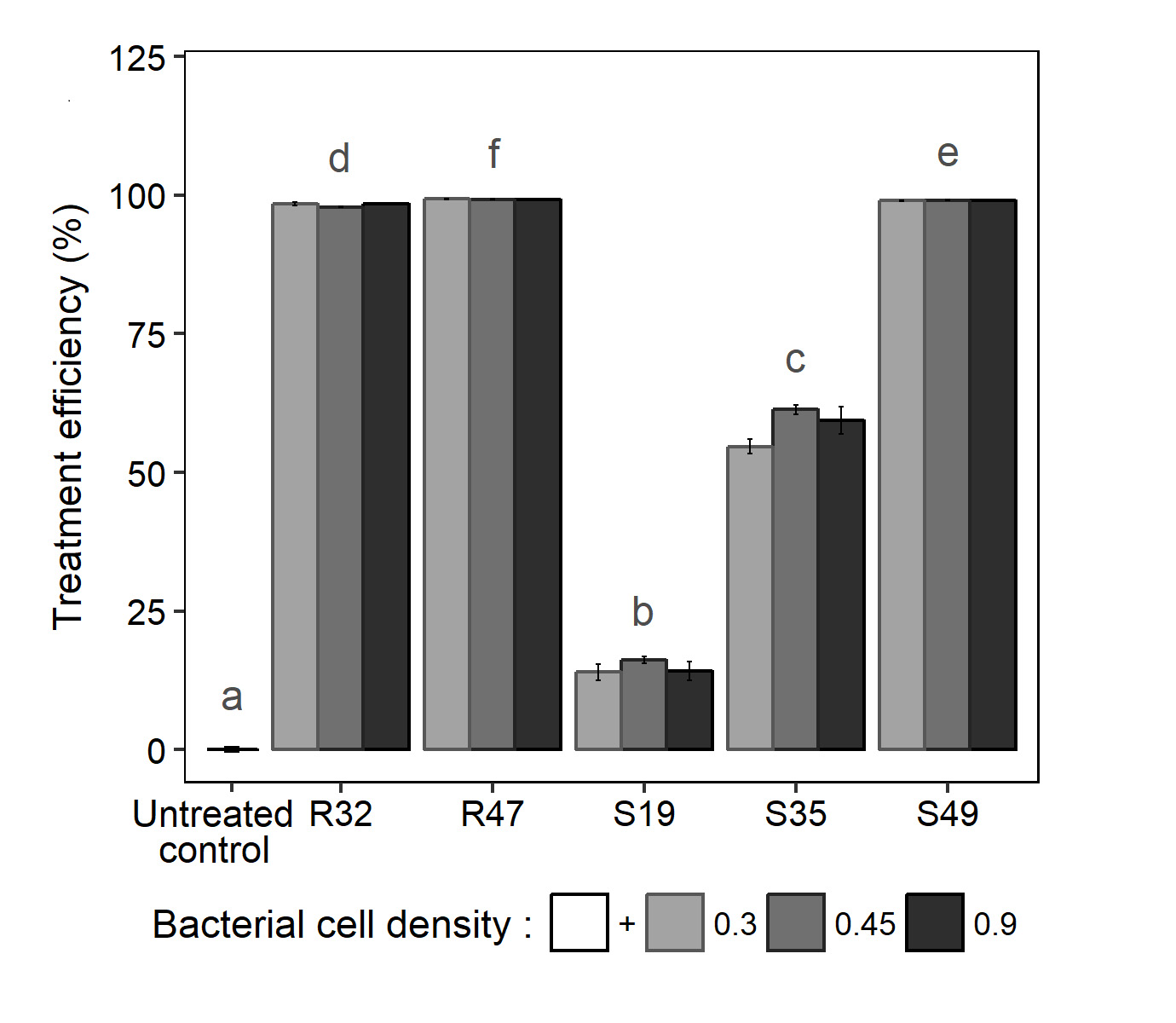

Supplement: FIGURE S2 — Relative mycelial growth of P. infestans when exposed to five Pseudomonas applied as single strains at three different bacterial cell densities in a dual culture Petri dish assay. Untreated controls represent P. infestans grown without bacteria (see Materials and Methods for details). Relative mycelial growth was calculated by dividing the mycelial area obtained in the respective treatments with that obtained in the untreated controls (not exposed to bacteria). Results are means of four replicates from the same experiment. They are expressed as treatment efficiency and calculated as above (100 – relative mycelial growth). Letters indicate significant differences between treatments according to Kruskal–Wallis test (p < 0.05, n = 4). [file Image_2.JPEG]

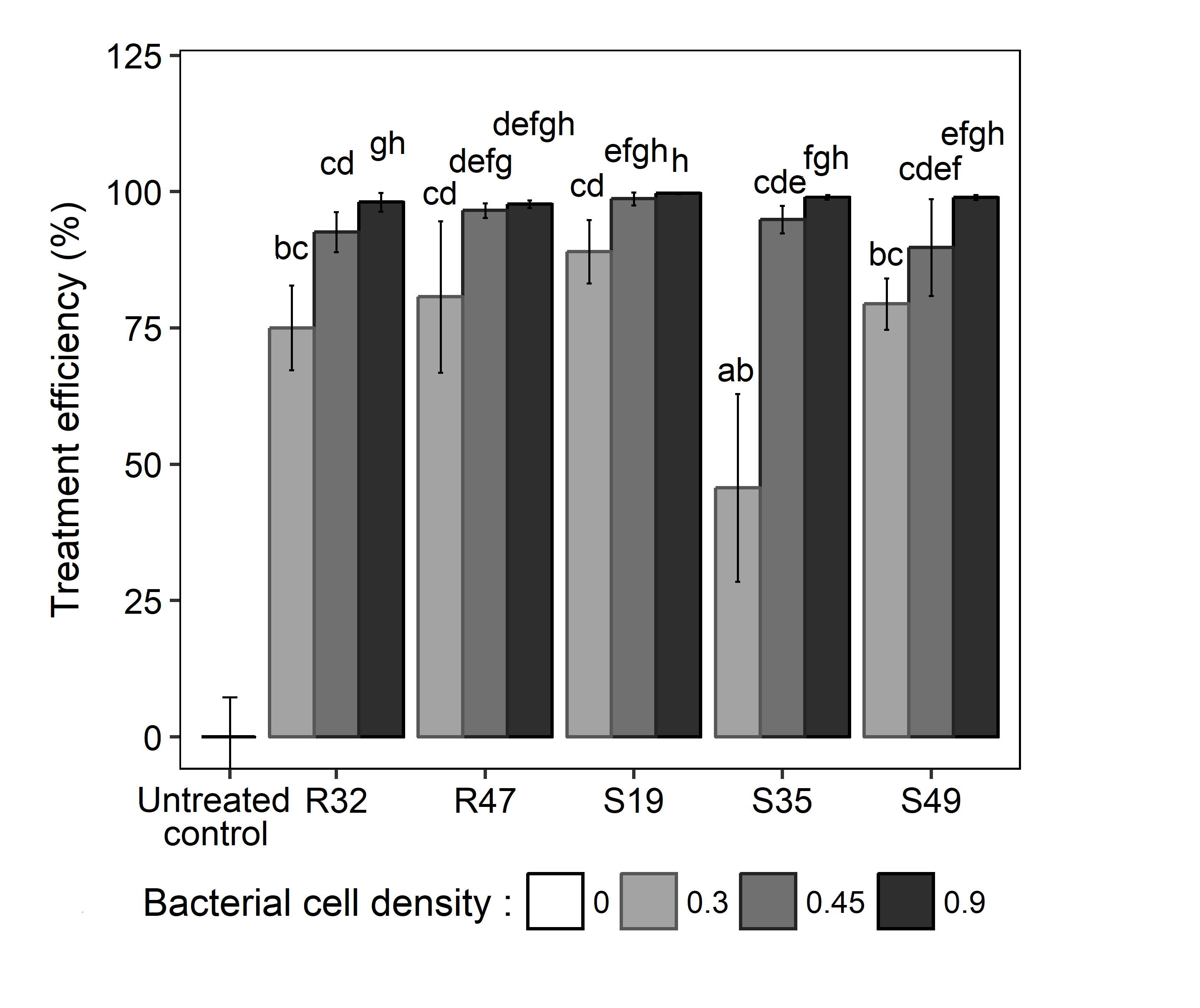

Supplement: FIGURE S3 — Relative zoospore release from P. infestans sporangia exposed to five Pseudomonas strains applied at three different cell densities. Sporangia suspensions pre-mixed with saline instead of bacterial cell solution were used as untreated controls. Released zoospores were counted and the relative release rate was calculated by dividing values obtained for treatments by those obtained for the untreated controls (see Materials and Methods for details). Results are means of three experiments with one sample per treatment. They are expressed as treatment efficiency and calculated as above (100 – relative zoospore release). Letters indicate significant differences between treatments according to Kruskal–Wallis test (p < 0.05, n = 3). [file Image_3.JPEG]
